# Cyclic tensile tests of Shetland pony superficial digital flexor tendons (SDFTs) with an optimized cryo-clamp combined with biplanar high-speed fluoroscopy

**DOI:** 10.1186/s12917-021-02914-w

**Published:** 2021-06-25

**Authors:** Franziska C. Wagner, Sven Reese, Kerstin Gerlach, Peter Böttcher, Christoph K. W. Mülling

**Affiliations:** 1 Institute of Veterinary Anatomy, Histology and Embryology, Faculty of Veterinary Medicine, Leipzig University, An den Tierkliniken 43, 04103 Leipzig, Germany; 2 Chair of Anatomy, Histology and Embryology, Department of Veterinary Sciences, LMU Munich, Veterinärstraße 13, 80539 Munich, Germany; 3 Department for Horses, Faculty of Veterinary Medicine, Leipzig University, An den Tierkliniken 21, 04103 Leipzig, Germany; 4 Small Animal Clinic, Department of Veterinary Medicine, Freie Universität Berlin, Oertzenweg 19 b, 14163 Berlin, Germany

**Keywords:** Equine, Horse, Superficial digital flexor tendon (SDFT), Cryo-clamp, Strain, XROMM

## Abstract

**Background:**

Long-term cyclic tensile testing with equine palmar/plantar tendons have not yet been performed due to problems in fixing equine tendons securely and loading them cyclically. It is well established that the biomechanical response of tendons varies during cyclic loading over time. The aim of this study was to develop a clamping device that enables repetitive cyclic tensile testing of equine superficial digital flexor tendon for at least 60 loading cycles and for 5 min.

**Results:**

A novel cryo-clamp was developed and built. Healthy and collagenase-treated pony SDFTs were mounted in the custom-made cryo-clamp for the proximal tendon end and a special clamping device for the short pastern bone (os coronale). Simultaneously with tensile testing, we used a biplanar high-speed fluoroscopy system (FluoKin) to track tendon movement. The FluoKin system was additionally validated in precision measurements. During the cyclic tensile tests of the SDFTs, the average maximal force measured was 325 N and 953 N for a length variation of 2 and 4 % respectively. The resulting stress averaged 16 MPa and 48 MPa respectively, while the modulus of elasticity was 828 MPa and 1212 MPa respectively. Length variation of the metacarpal region was, on average, 4.87 % higher after incubation with collagenase. The precision of the FluoKin tracking was 0.0377 mm, defined as the standard deviation of pairwise intermarker distances embedded in rigid bodies. The systems accuracy was 0.0287 mm, which is the difference between the machined and mean measured distance.

**Conclusion:**

In this study, a good performing clamping technique for equine tendons under repetitive cyclic loading conditions is described. The presented cryo-clamps were tested up to 50 min duration and up to the machine maximal capacity of 10 kN. With the possibility of repetitive loading a stabilization of the time-force-curve and changes of hysteresis and creep became obvious after a dozen cycles, which underlines the necessity of repetitive cyclical testing. Furthermore, biplanar high-speed fluoroscopy seems an appropriate and highly precise measurement tool for analysis of tendon behaviour under repetitive load in equine SDFTs.

**Supplementary Information:**

The online version contains supplementary material available at 10.1186/s12917-021-02914-w.

## Background

The superficial digital flexor tendon (SDFT) is one of the most frequently injured structures of the musculoskeletal system in sport horses, particularly in the forelimb [1–4]. Due to its function, the equine SDFT works close to its physiological limit under maximal load [5]. Loads of up to 14.5 kN [6] and strains of up to 16.6% have been reported during gallop [7].

Long-term cyclic tensile tests have not yet been performed due to insufficient clamping techniques. Riemersma and Schamhardt [8] proposed a cryo-jaw for tensile testing of large animal tendons, which was used and further developed by other investigators [9–12]. Cryo-jaws have the advantage of holding all tendon fibres equally, without squeezing them, whereas mechanical compressive clamps squeeze the cellular water, which results in slippage [8]. Cryo-clamps have been used to hold equine digital flexor tendons (DFT) during rupture or failure tests [8, 13–16, 9]. However, during repetitive loading, problems arose including: thawing of the tendon, slipping out of the clamp during cyclic testing [11], breakage of the frozen tendon at the clamp exit. Thus, this limits the possible number of testing cycles. A cyclical loading of equine DFT, up to 3 kN with 30 cycles, was achieved by Swanstrom et al. [17] by fixating the elbow and phalanges with bone pins. Thorpe et al. [15], also with a cryo-clamp, performed 20 loading cycles of up to 4 kN.

In the current study we aimed to develop a clamping device for tendon testing of up to 10 kN and for repetitive tensile testing with at least 60 loading cycles, combining the advantages of former devices. Additionally, we used 3D X-ray motion analysis (fluoroscopic kinematography, FluoKin) to measure the tendon strain of the equine SDFTs via implanted tantalum beads. FluoKin operates as a biplanar, high-speed, fluoroscopic XROMM system [18] with a reported high precision (0.12 ± 0.08 mm and 0.09 ± 0.08° [19]) that can also be used to detect tendon motion [20]. From calculations based on Tashman and Anderst [21], we expected a tendon length variation of about 4 mm to 8 mm during simulated walk and trot.

The presented clamping device was used to reliably analyse healthy and collagenase-injured tendons movement during simulated walk and trot loading. Additionally, precision of the fluoroscopic gait lab was determined to establish high-speed fluoroscopy of the equine SDFT.

## Results

### Tensile tests

**Fig. 1:**
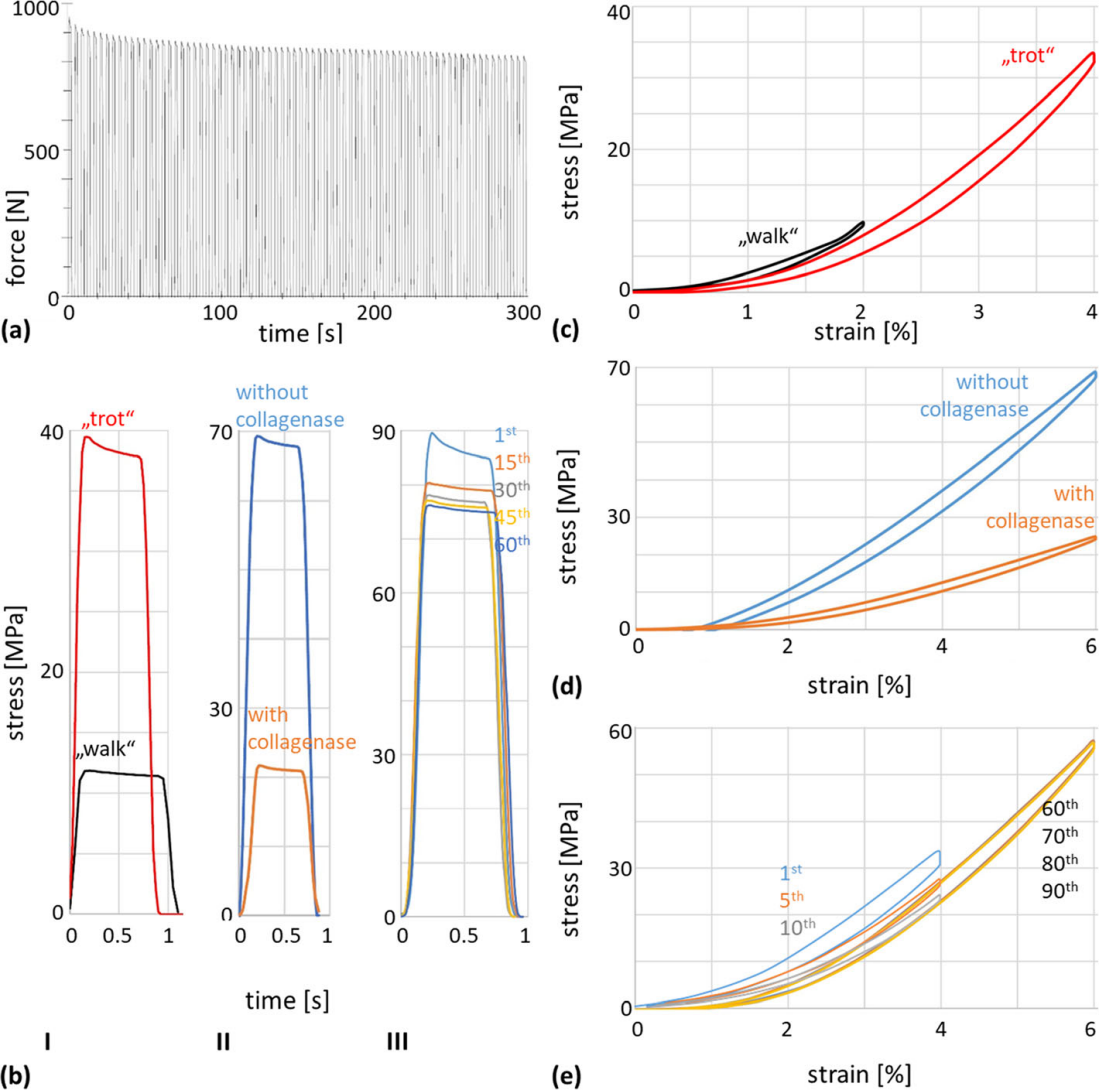
Loading and unloading patterns of Shetland pony SDFT. **a** Typical force-time-diagram of repetitive cyclic tensile testing at 4% strain with initial decrease of force and plateau phase., **b** Stress-time-curves normalized over 1 s, **b (I)** creep effect in the 1st cycle at 2 and 4% strain, **b (II)** creep effect in the 1st cycle at 6% strain in untreated SDFT (blue) and with collagenase (orange) during the 60th cycle, **b (III)** creep effect during the 1st to 60th cycle in untreated SDFT at 6% strain, **c** Hysteresis curves during “walk” (black) vs. “trot” (red) during the 60th cycle, **d** Hysteresis curves in untreated SDFT (blue) and with collagenase (orange) during the 60th cycle, **e** Hysteresis curves at 4% strain (“trot”) during the 1st to 10th cycle and at 6% strain during the 60th to 90th cycle in untreated SDFT

**Fig. 2:**
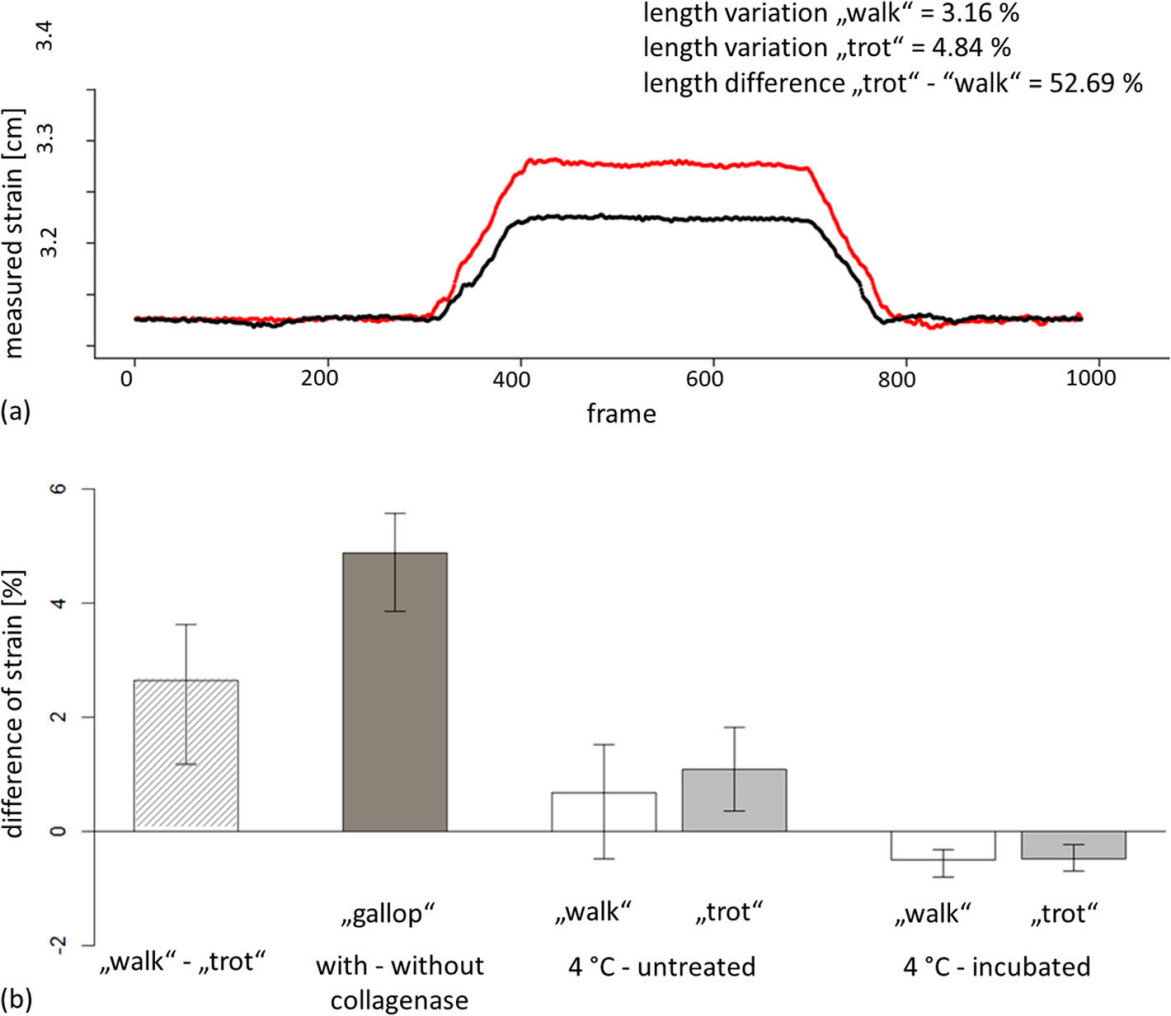
Measuring strain of the SDFT with FluoKin. Measuring strain within the 60th loading/unloading cycle on the basis of changing intermarker distances of implanted tantalum beads in the SDFT detected with FluoKin. **a** Spatial displacement of the proximal and distal tantalum bead at walk (black) and trot (red), **b** Mean difference between two tantalum beads during load cycles mimicking different gaits (error bars reflecting its maximum and minimum), dark grey: “gallop”, white: “walk”, grey: “trot”

Isolated SDFTs of Shetland ponies, with implanted tantalum beads, were strained in a loading machine and simultaneously video-radiographed with FluoKin. Biplanar video-radiographs with FluoKin were taken within the 60th cycle, when the force to strain the tendon reached a plateau and the tendons were in a preconditioned status (Fig. 1 a). During tensile testing all tantalum beads remained in their position, even after injection of collagenase. A difference in tendon lengths between “walk” and “trot “was significantly detected (*p* < 0.025) and ranged from about 1.17 cm to 3.83 cm (Fig. 2 a). A difference in tendon lengths could also be shown in the two foal tendons tested in “walk”, “trot”, and “gallop”. Untreated tendons also differed from those injected with collagenase (*p* < 0.002), ranging from about 3.86 cm to 5.57 cm. There was no significant difference between incubated tendons and those kept at 4 °C overnight (Fig. 2 b).

To lengthen the SDFTs by 2%, 325 N were necessary on average, whereas 953 N were required to achieve a lengthening of 4%. Thus, an average stress of 16 MPa and 48 MPa, and an average modulus of elasticity of 828 MPa and 1212 MPa, resulted respectively for 2 and 4% lengthening (Table 1). The tendon strain and modulus of elasticity were reproducible after 30 min of rest.

**Table 1:** Mechanical properties of the SDFTs of mature Shetland ponies (data from 21 measurements)

	F _max_ [N]	Stress [MPa]	Modulus [MPa]
„walk“	189–497 mean: 325 ± 74	11–23 mean: 16 ± 3.5	551–1176 mean: 828 ± 177
„trot“	537–1300 mean: 953 ± 166	34–61 mean: 48 ± 7.2	860–1523 mean: 1212 ± 179

When plotting the data of stress and strain at the 60th test cycle, continuously over time, the relaxation curve is lower than the loading curve. This phenomenon is called hysteresis. Simulating “trot” we found a pronounced cushioning effect, whereas this effect was very low when simulating “walk” (Fig. 1 c). After injection of collagenase the hysteresis curve rose much slower and the area between the loading and relaxing curve is decreased by 17.2 -55.2% (Fig. 1 d). The hysteresis curves of the 60th to 90th loading cycle were almost completely congruent (Fig. 1 e).

During the short holding phase with constant strain of each cycle, when the tendon is loaded, the stress [MPa] decreased significantly at 2% strain (“walk”) 0.029 ± 0.008%, at 4% strain (“trot”) 0.048 ± 0.018%, and at 6% strain (“gallop”) without collagenase 0.086 ± 0.004%, and with collagenase 0.121 ± 0.013% (Fig. 1 b). This phenomenon describes the “creep”. The effect of creep was more pronounced in the early loading cycles and attenuated steadily (Fig. 1 b).

To test the capacity of the clamps to hold the tendon, a repeated cyclic loading with at least 50 min duration at 1.5 kN was carried out with the cryo-clamps for Shetland pony SDFT. Additionally, failure test until rupture were carried out. No slippage of the tendon was observable or other signs of failure (Fig. 1 a and Additional file 2).

### Precision measurements of the FluoKin system

**Fig. 3:**
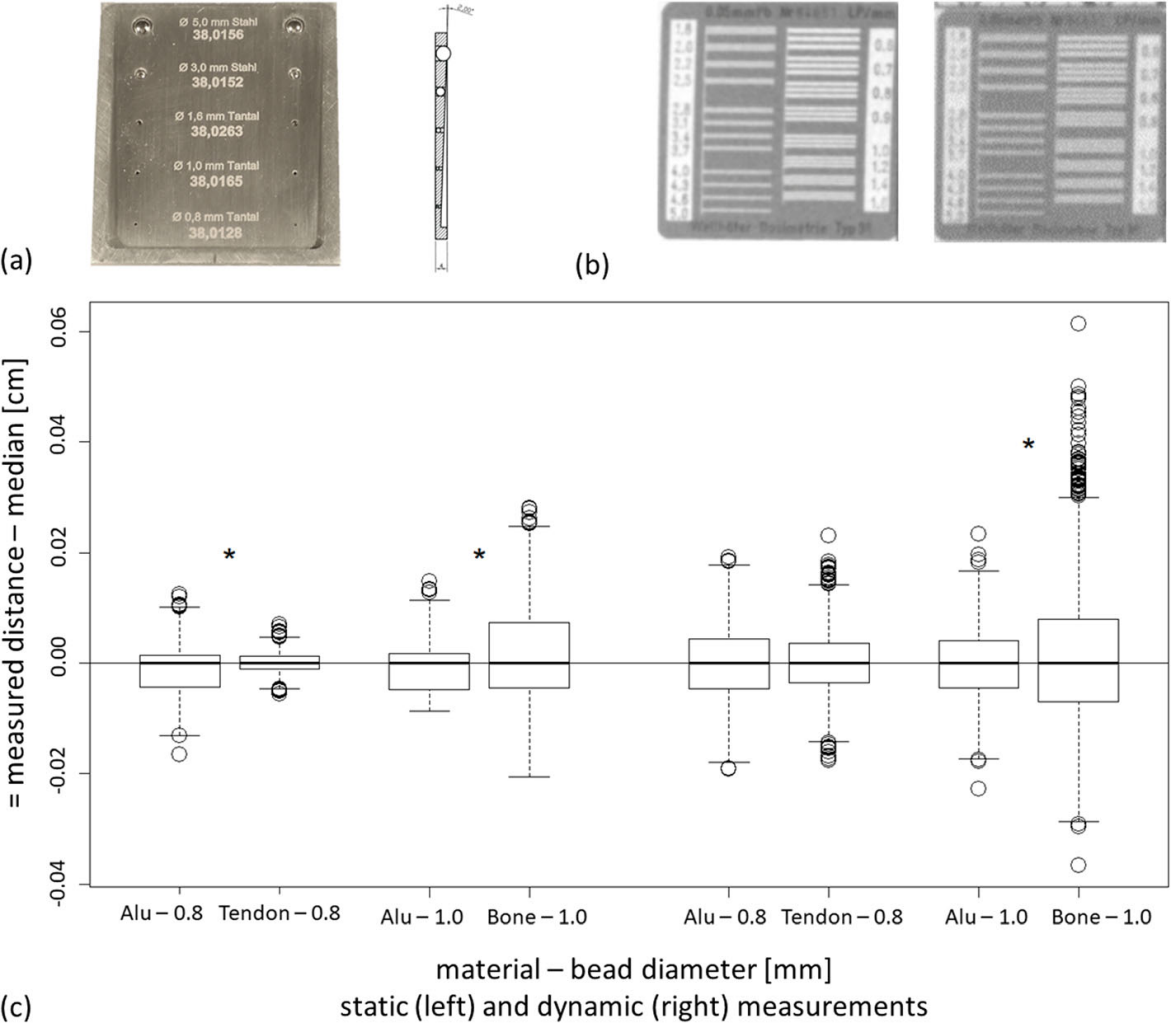
Measuring precision of the FluoKin gait lab. **a** Test sheet (70 mm × 60 mm × 4 mm to 2 mm falling edge from the largest to the smallest beads, front view (with imprinted distance [mm] for each pair of beads) and side view), **b** Resolution of the FluoKin system, images of the two image intensifier/camera systems without magnification, **c** Precision measurements with tantalum beads (0.8 mm and 1.0 mm) in an aluminium sheet and a forelimb (tendon and bone); *significant difference (*p* < 0.019)

To determine accuracy and precision of the FluoKin lab an aluminium sheet with embedded beads was imaged in a static position and by dynamically waving it through the image field (Fig. 3 a and c, Table 2). The mean measured distance between all bead pairs differed significantly between the static and dynamic measurement (*p* < 0.0001).

**Table 2:** Characteristics of precision and accuracy of the FluoKin gait lab. Static (stat) and dynamic (dyn) measurements with implanted beads in an aluminium sheet (mean values)

implanted beads	distance in micro-CT ±0.0003 [mm]	mean measured distance [mm]		mean absolute error [mm]		accuracy [mm]		precision [mm]		range [mm]	
		stat	dyn	stat	dyn	stat	dyn	stat	dyn	stat	dyn
0.8 mm tantalum beads	38.0128	38.071*	38.056*	0.0617	0.0627	0.0586	0.0432	0.0431	0.0645	0.2891	0.3836
1.0 mm tantalum beads	38.0165	38.045*	38.065*	0.0424	0.0660	0.0287	0.0489	0.0444	0.0610	0.2348	0.4605
1.6 mm tantalum beads	38.0263	38.090*	38.076*	0.0657	0.0655	0.0640	0.0501	0.0377	0.0641	0.2639	0.3832
3.0 mm steel beads	38.0152	38.123*	38.065*	0.0108	0.0655	0.1076	0.0503	0.0376	0.0636	0.2571	0.4249
5.0 mm steel beads	38.0156	38.151*	38.066*	0.0135	0.0757	0.1349	0.0503	0.0390	0.0795	0.2617	0.5357

* significant difference to machined distance (*p* < 0.0001)

The 0.8 mm and 1.0 mm tantalum beads were also implanted in the SDFT and metacarpal bone respectively, of a forelimb. The limb was then frozen to prevent any marker movement during static and dynamic measurements (Table 3).

**Table 3:** Precision of the FluoKin gait lab measured with implanted beads in a frozen forelimb

	Precision [mm]		Range [mm]		Precision [°]	
	static	dynamic	static	dynamic	static	dynamic
Tendon markers (0.8 mm tantalum beads)	0.0144 *	0.0553 #	0.0377	0.2779	0.06	0.14
Bone markers (1.0 mm tantalum beads)	0.0562 *	0.0985 #	0.5288	0.9753	0.18	0.26

* and # precision between tendon and bone markers differs significantly between static and dynamic measurements (p < 0.0001)

Spatial resolution of the FluoKin system (including the two image intensifiers, lens system, and high-speed cameras) was 1.2 lp/mm and 1.0 lp/mm in standard mode, 1.8 lp/mm and 1.4 lp/mm in magnification mode 1, and 2.2 lp/mm and 2.0 lp/mm in magnification mode 2 (Fig. 3 b).

## Discussion

In the present study, we developed a good working cryo-clamp for prolonged repetitive cyclic tensile testing with equine SDFT. This surpasses the previous problems in fixing equine tendons securely during cyclic testing. The results of our tensile testing with Shetland pony SDFTs suggests that measuring biomechanical properties should be undertaken in properly preconditioned tendon tissue when the plateau phase is reached. Additionally, length variation of moving tendons was recorded with FluoKin, a biplanar X-ray analysis system, through tracking position changes of implanted tantalum beads. Furthermore, the precision of the FluoKin system was assessed. We will now provide a detailed description of the clamp, discuss the results of the tensile testing, and end with discussion about the precision testing.

### An optimized cryo-clamp for equine tendons

**Fig. 4:**
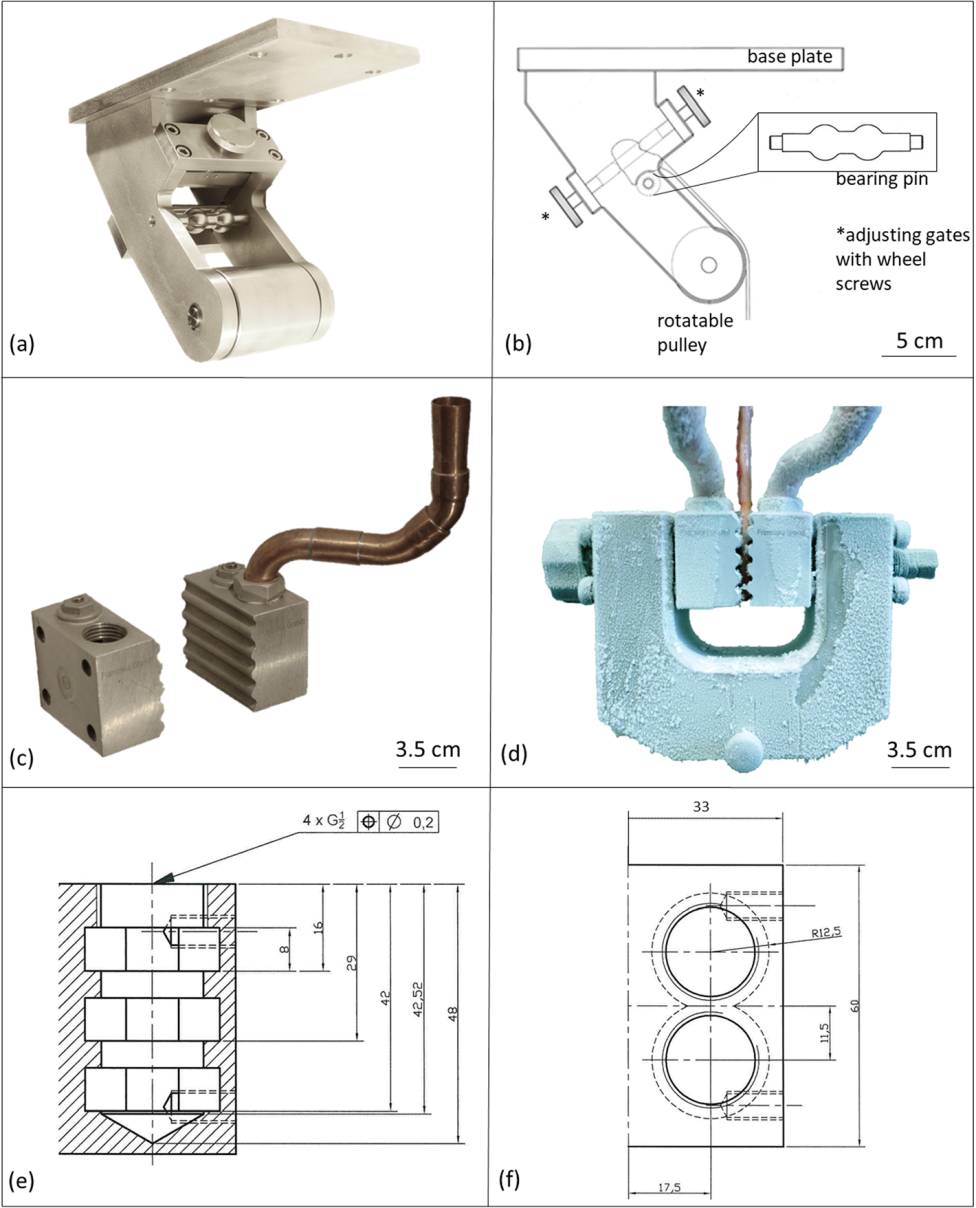
Mounting and cryogenic clamping devices for cyclic testing with pony SDFT. **a** mounting device for the short pastern bone (os coronale) with **b** technical drawing including a schematic of the specimen, **c** cryo-clamp, opened and with copper filling tube, **d** SDFT in the cryo-clamp mounted in the u-shaped connector, **e** technical drawing of one cryo-jaw, side view, **f** technical drawing of one cryo-jaw, top view

In 1982, Riemersma and Schamhardt proposed a cryo-jaw for testing equine digital flexor tendons. This design has been modified over the years [9, 22–25]. Long-term cyclic tensile tests have not been performed so far due to problems such as thawing of the tendon and slipping out of the clamp [11]. As Fig. 1 a shows, there is a drop in the required force, especially in the first loading cycles. Therefore, it is recommended to take measurements only after the force-time curve has stabilized and the tendon is in a preconditioned status. As part of the work presented here, we designed and built a cryo-jaw for prolonged cyclic tensile tests that avoids slippage, damage, or disruption of the tendon (Fig. 4 c).

The hollow cryo-clamps were filled with liquid nitrogen. Vents in each clamping jaw prevented pressure from building up. The filling tubes were made of copper, making it easy to curve and process them. Rubber ring seals prevented the filling tubes of slipping out of the clamp. The funnels for filling the liquid nitrogen into the copper tubes were made of Teflon, a synthetic material that is resistant to − 200 °C. Freezing the tendon on the clamp by cooling it with liquid nitrogen attached all tendon fibres equally to the clamp. It seemed essential, for repetitive cyclic testing, to keep the clamp cold continuously. Otherwise, slippage is likely above 3.5 kN [8]. A wave profile increased the contact surface providing a large freezing area. The clamp profile was adapted to the thickness of pony SDFTs. Its outline enabled closure of the clamping jaws without shear forces occurring, because of an even distance between the two profiles at every distance of the two clamping jaws. We recommend using a clamp profile adapted to the tendon’s diameter to completely freeze and securely hold it. In this way, the freezing and special wave profile ensured that all tendon fibres are included in the loading during tensile tests which imitated physiological conditions more closely. Unlike mechanical clamps, there are no artefacts due to squeezing. To prevent any disruption of the deep-frozen tendon at the edges of the clamps, where the tissue exited, were radiused at 2 mm and the clamps offset from one another by 10°.

Breakage of the tendon tissue above the clamps or slippage out of clamp did not occur at 10 kN, which was the maximum capacity of the testing machine. This quality feature of the cryo-clamps would also allow the performance of yield and rupture tests (see Additional file 2). With Shetland pony SDFTs, up to 50 min cyclic tensile testing with 1.5 kN was performed without tendon slippage out of the clamp. At that point, the liquid nitrogen stock was used up. Experiments with longer duration and higher loads are feasible. Nevertheless, to ensure proper testing, storing the clamps at − 80 °C overnight prior to the experiment is recommended. Additional cooling by a sleeve with dry ice around the cryo-clamps could reduce the consumption of liquid nitrogen. Aluminium, as a clamping material, ensures a high thermal conductivity and sufficient resistance. The clamps surface that was in contact with the tendon was roughened for an optimal temperature transfer. We measured the temperature gradient along the fixated tendon with an infrared thermometer. We measured − 7 °C on the tendon surface next to the cryo-clamp. The surface of the tendon tissue of all specimens reached room temperature at the longest 3 cm away from the frozen clamp. Similar cryo-clamps have required a safety distance of 5–6 cm from the cryo-clamps to the measuring zone [8, 14, 16]. In order to avoid cold artefacts within the measuring zone the entire length of the tendon should be used.

**Fig. 5:**
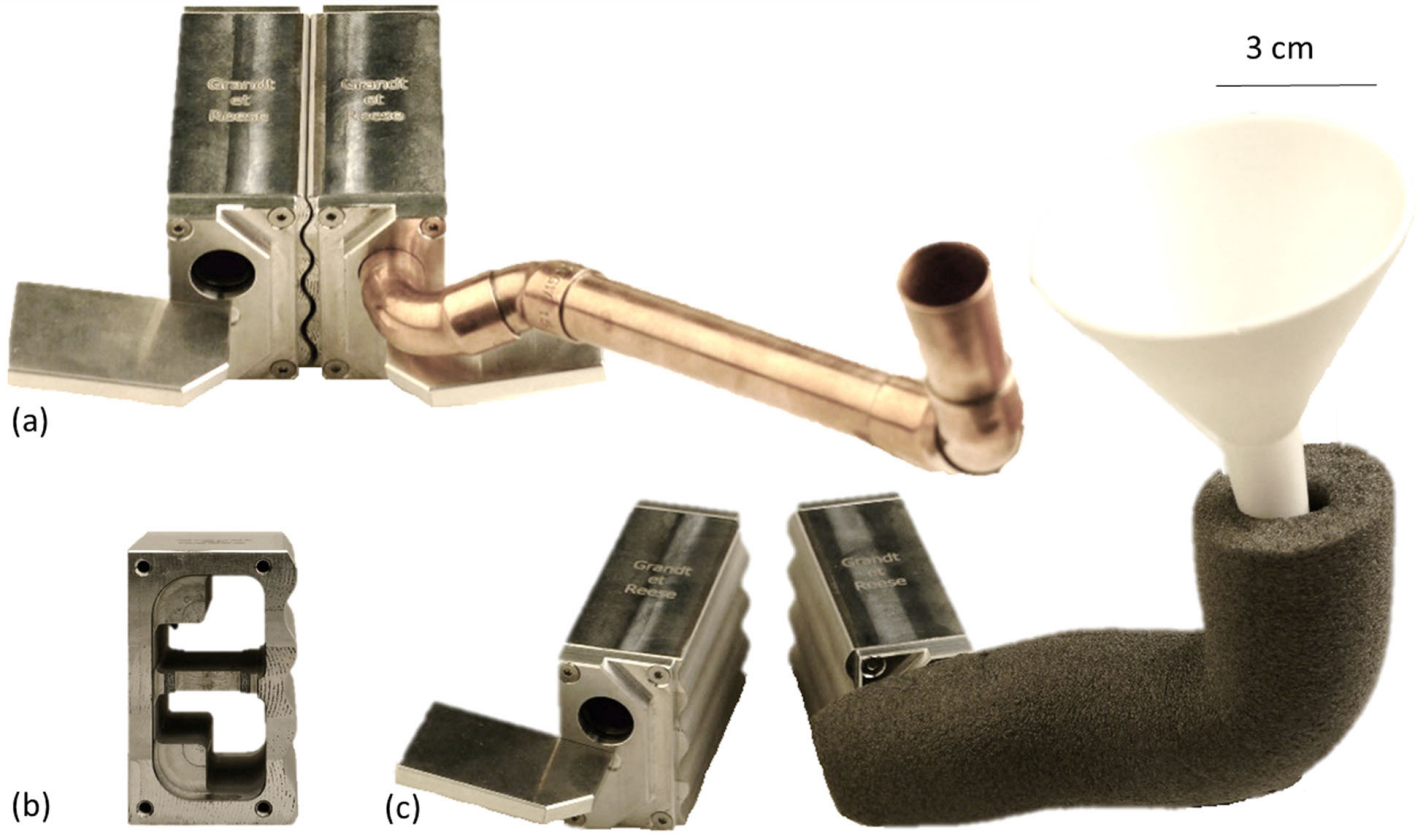
Cryogenic clamping device for **pony DDFT or SDFT of horses** (30 × 55 × 80 mm). **a** Front view with copper filling tube, **b** Inside front view, **c** Front view with coated filling tube and Teflon funnel. We used a custom foam pipe insulation to keep the clamping device cold for a noticeably longer time

The cryo-clamp described here may not be appropriate to perform repetitive cyclic tensile tests with tendons containing a larger diameter than the SDFTs of ponies. To test the SDFTs of horses or the DDFTs of ponies, which have larger diameter tendons, we built another cryo-clamp with a larger clamping profile (Fig. 5). Its cooling chamber is also more spacious, because the clamp as a whole is larger. To create a larger imaging field in FluoKin, the filling tubes of the larger clamp enter frontally. This enables an automatic tracking of the tantalum beads implanted in the tendons. Also, the tendon was strained with a previously unreached repetitively applied, tensile force of 9500 N. This was limited not by the cryo-clamp itself but rather by the maximum force of the machine (10 kN).

The proximal end of the SDFTs were fixated in the cryo-clamp, whereas to hold the distal end of the SDFTs, the enthesis of the short pastern bone (os coronale) was used. Using the attached bone to hold the tendon provided advantages for occupational safety. More specifically, no liquid nitrogen cooled cryo-clamp was needed at the moving transverse of the tensile testing machine. During the tensile tests, the curved shape of the bearing pin distributed the force at the articular surface of the os coronale better than a simple rod shape. During tensile stress, force is also built up on the os coronale via the palmar adjusting gate. To minimize force peaks and to enlarge the contact area, the adjusting gate was placed at the height of an anatomical indentation at the palmar aspect of the os coronale.

Though the SDFT undergoes tensile loading throughout the whole stance phase, the peak load occurs in the early stance phase [26]. The device imitated the physiological position of the os coronale when the leg stands vertically through an angulation of 55°. Additionally, the position of the os coronale could be fixed with two adjusting gates, which are in grooves and shifted via wheel screws. We imitated the gliding surface of the scutum proximale with a rotatable cylinder. Consequently, during tensile testing, the tendon fibres were loaded in their physiological force direction of the main load phase. The parts of the device were screwed together, rather than welded, for better force resistance. The stainless steel used provided corrosion resistance.

A limitation of the mounting device was its fixed angles. To better imitate a step, a tiltable device would be beneficial. To also test the SDFTs of horses, the device would need to be larger. Also, a slidable pulley would allow for a more suitable adjustment. For repetitive cyclic tensile testing we recommend using this proposed novel mounting device for the bone, with the tendon’s enthesis, in order to avoid the need for a second cryo-clamp. Additionally, the tendon fibers can be loaded in their natural direction.

### Tensile tests

To test the new cryo-clamp Shetland pony SDFTs were subjected to repetitive cyclic tensile testing and failure tests until rupture. The tensile testing protocol contained loading, loaded, unloading and unloaded phases with strain and time dependent on the imitated “gait”. We decided to use 2, 4 and 6% for “walk”, “trot” and “gallop” respectively. These values were estimated for the body weight of Shetland ponies based on Riemersma et al. [21] who found a lengthening in vivo of 2.13–2.19% during walk and 3.99–4.15% during trot in the SDFTs of Icelandic ponies. In horses, Butcher et al. [1] reported 3.6% at walk, 5.6% at trot, and 4.8% at canter, also in the SDFTs. Those values seem to be dependent on the gait velocity, e.g. Lawson et al. [27] found 6.7% at walk and 8.5% at trot. Strain of the SDFTs of Shetland ponies in vivo still needs to be determined and related research is underway.

For more reproducible measurements, it is recommended to precondition the tendon by loading it 10 to 30 times prior to measuring [28–30]. Preconditioning implies a reversible change of the microstructural response to strain [31] such as lengthening of the fibres [32] through straightening of the fibre’s crimp and squeezing out the procollagen water mixture [33–35, 31]. Video-radiographs in our study were taken during the 60th cycle to ensure we measured within the plateau phase of the force straining the tendon. Dakin et al. [10] tested several repetitions after a single cycle of preconditioning. They strained the SDFTs up to 5% with 0.5 Hz. Swanstrom et al. [17] tested equine DFT 30 times with 1 Hz for 400 N to 3000 N after 5 cycles of preconditioning. Thorpe et al. [36] described tensile tests of the equine SDFTs up to 4 kN with 20 repetitive cycles. To the authors best knowledge there have not been any other prolonged cyclic tensile tests with equine SDFTs to date.

In the current study with Shetland pony SDFTs, 325 N and 953 N on average were required on average to lengthen the specimens of 2 and 4% respectively. In pony tendons 2.2–5.5 N/kg at walk and 6.3–7.0 N/kg at trot were measured in vivo [26, 37, 38, 21]. We used tendons of Shetland ponies (estimated body mass of about 130–180 kg) in our study which would result in a force of 1.05–1.45 N/kg at “walk” and 7.2–10.0 N/kg at “trot”. Measured in horses in vivo, the average maximum force has been reported to be 3.1–3.8 kN at walk, 4.7–6.0 kN at trot and 5.1–7.0 kN at canter [39]. Ex vivo, maximum forces in horses ranged between 6.7–14.5 kN [13, 6, 40, 11, 14, 15].

Stress, measured in the present study with Shetland pony tendons, was higher (11–61 MPa) than measured previously in horses (45.7–71.8 MPa [1, 13, 11];), due to their smaller cross-sectional area (0.21 ± 0.04 cm^2^). For Shetland ponies, the elastic modulus in our study was between 551 and 1523 MPa. Elastic modulus ex vivo has been suggested to vary between 740 and 1408 MPa in horses [13, 6, 40, 17, 15].

Plotting the loading and unloading curves of “walk” and “trot” hysteresis is obvious (Fig. 1 c). Hysteresis refers to an energy dissipation due to viscoelastic properties and is described by the area and distance between the two curves [41]. Hysteresis correlates with the absorption of energy [41] and the scope of the cushioning effect [42]. During “trot” the tendon undergoes higher stress than during “walk” and more hysteresis can be seen (Fig. 1 c). Simulating “trot” forces the tendon fibres to strain more viscoelastically, rather than elastically. The higher the utilization of elastic energy the lower is the hysteresis [43–45]. In contrast to our ex vivo simulations of “walk” and “trot”, hysteresis was minimized at high strain rates [46] in order to optimize energy efficient locomotion in vivo. The hysteresis effect reached a constant phase between the 60th and 90th loading cycle indicating that the tendon tissue is in a preconditioned status (Fig. 1 e).

During the short holding phase with constant strain, within each cycle a significant decrease of stress was detected (Fig. 1 b). This phenomenon is called “creep” or “cold flow”. Simulating trot, this decrease was significantly more pronounced than at walk (*p* = 0.018). During 6% strain, the stress decreased significantly more in the collagenase-treated tendons than in those without collagenase (*p* = 0.034). The creep occurred mainly in the first third of the holding phase (Fig. 1 b). During this phase, energy stored within the tendon is released, which is advantageous for absorbing vibrations [42]. This viscoelastic behaviour of the SDFT is one of its cushioning mechanisms. The effect of creep attenuated as the number of loading cycles increased (Fig. 1 b III), which is also a sign that the tendon tissue has reached a preconditioned status.

In collagenase-treated tendons Nabeshima et al. [47] found a decrease of stiffness, maximum failure force, and both elongation and energy to maximum force. Crevier-Denoix et al. [40] compared healthy and injured tendons and found a decrease of maximum load of 1.4 kN and maximal stress of 71.3 MPa, on average. Our results are in accordance with those of Crevier-Denoix et al. [6] who also needed higher force to strain injured tendons. Length variation measured with FluoKin was on average 4.87% higher in specimens treated with collagenase than without (Fig. 2 b). The massive changes in the biomechanical properties of the SDFTs after injection of collagenase can also be seen in the hysteresis curve. Figure 1 d shows this as less uptake of stress, a smaller area between the curves, and as a higher creep (Fig. 1 b). Collagenase-induced degradation of the tendon tissue and structure of the collagen network (e.g. [48, 49]) likely leads to the changes of the tendon’s biomechanical response. The destruction of collagen fibres by collagenase leads to less resistance to tensile force, less absorption of energy and to a decrease of the cushioning effect. The interaction of the collagen fibres in processes such as interfibrillary cross-linking or electrostatic attraction [50, 51] is disturbed through the disruption of their structural properties.

Using a Clostridium histolyticum derived collagenase is common to initiate a tendon lesion [52–55, 47] but limits our conclusions because of its multiple action sites along the collagen triple-helix compared to mammalian collagenase, which reacts only at a single site [48, 56]. Additionally, ex vivo testing lacks the inflammatory response and a functioning cell-to-cell-communication. Nevertheless, our results show that collagenase treatment of equine SDFTs ex vivo was able to influence the biomechanical response to strain and provide useful information for in vivo experiments.

The present study focused on the forelimb SDFTs of the Shetland pony. Its energy storing ability depends on the gait speed, with stress increasing with speed, whereas this ability remains more constant in the hindlimb [57]. Riemersma and Schamhardt [58] found an elastic modulus of 1000–1282 MPa in the hindlimb SDFTs. The hindlimb’s functional specialization could lead to different mechanical characteristics of its SDFTs.

Differences in the biomechanical behaviour of the tendons between pony and horse are possible. A pony’s hoof is relatively small with high heels and a short toe. Additionally, the propulsion phase during stance is less ample compared to horses [59]. Deviation due to sex, age, race, training, ground surface, anatomical site, and method of measurement and its precision are also known to affect the tendon’s biomechanical response. A difference in age and sex of the donor animals might also be a reason for the relative broad ranges of maximum force, stress, and modulus of elasticity in Table 1.

Age-dependent changes in biomechanical behaviour of tendons due to morphological and biochemical changes have been widely studied [60–66]. We did not notice any significant age-related influence on our results (with the exception of the two specimens derived from foals). Some studies in humans report a decrease of tendon stiffness with age [67, 68], while others report an increase [69]. Addis and Lawson [60] found an increase of strain with age. Thorpe et al. [65] found no influence of age on elastic modulus, failure stress, or strain of the whole SDFTs, but a significantly less fascicle sliding in aged tendons.

In the current study, load-dependent length variation in the SDFTs was detected for the first time with biplanar high-speed fluoroscopy. During the experiments the position of the implanted tantalum beads remained stable. As Smith et al. [70] commented, it is important to inject the beads across the grain to prevent bead migration along the injection canal in the tensile direction. One way to overcome this problem is also to drill a hole in the bead and suture it to the tissue [71] or to close the injection hole with fibrin glue [72, 73]. In vivo, tantalum is bio-inert [74] and can be used in long-term studies without tissue reaction.

We focused on the metacarpal region of the SDFTs, nevertheless it is also possible to video-radiograph the whole tendon with FluoKin. However, due to the narrow field of biplanar measurement (approximatively 6000 cm^2^) the image sequences must be assembled from different heights depending of the length of the specimen. The detection method, using implanted tantalum beads, could also be employed on other structures, e. g. on the deep digital flexor tendon (DDFT). It is advisable to inject the markers from lateral or medial in the DDFT and not from palmar/plantar in proximodistal direction as conducted in this study.

### Precision measurements of the FluoKin system

In the current study, FluoKin, a 3D X-ray motion analysis system, was used to measure the length variation between tantalum beads implanted in the SDFT. Additionally, the precision of the FluoKin system was assessed by measuring the intermarker distance between radiopaque markers embedded in rigid bodies. The results provide not only the precision estimations for this study, but also for our FluoKin gait lab in general.

To measure precision of the Leipzig FluoKin gait lab, we used an aluminium sheet and a frozen distal forelimb with embedded markers which should remain in a stable position within rigid bodies [20]. To assess the accuracy of the system, a test object with a known distance between the markers is required [20]. In our study, all distances in the sheet measured with FluoKin differ significantly to the machined distance. Nevertheless, a qualitative conclusion cannot be drawn, because measurements taken with one technique are related among themselves, since they contain the same bias. Under optimal conditions, the Leipzig FluoKin gait lab can assess an accuracy of 0.0287 mm, a precision of 0.0377 mm and a range of 0.2348 mm. These values are in the same range as data from the Keck Foundation XROMM Facility at Brown University (precision of 0.046 mm, 18) and the 3D^2^YMOX system at the University of Antwerp (precision of 0.066 mm, range of 0.236 mm, [75]).

The quality of marker tracking depends on the diameter of the markers and the contrast to the surrounding material and can be reached under static conditions. Dynamic measurements are generally more imprecise than static ones due to the inevitable motion blur. X-ray permeability of tendon tissue is better than the more radiopaque bone tissue. It seems that beads with larger diameter are tracked more precisely (with lower standard deviation), but the accuracy of smaller beads more circumscribed in the X-ray image could not be reached. This study compares for the first time precision parameters of 5 different marker sizes within one FluoKin video. The highest precision was obtained using 0.8 mm beads in tendon tissue under static conditions (0.0144 mm). Whereas the most imprecise measurement occurred with 1.0 mm beads in bone tissue under dynamic conditions (0.0985 mm). In praxis, the choice of the bead diameter is often a compromise between precision parameters and enough contrast to the surrounding tissue. Additionally, to optimize contrast, the voltage of the X-ray tube can be suited to the *k*-absorption edge of the marker material [76], 90 kV should be chosen for tantalum [18]. The energy used should be a compromise between the best tantalum and tissue contrast. Software used for marker tracking evolves quickly and surely improves in terms of precision.

The main causes of imprecision are technical artefacts during image processing. To maximise precision all components from the X-ray hardware to the analysing software should be perfectly compatible with each other. For example, a loss of precision could be caused by one of the image intensifiers not producing an image of equal sharpness as the other, possibly due to its age. Image intensifiers convert the X-ray radiation into a visible and brightened light image. The spatial resolution of image intensifiers is typically about 4 lp/mm. We reached 1.0 to 2.2 lp/mm in standard and magnification mode. According to Brainerd et al. [18], the limitation in the image chain is the 1024 × 1024 pixels resolution of the high-speed cameras. Overall, our image quality, in terms of resolution, is comparable to that reported by Brainerd et al. [18] (1.5 lp/mm without magnification and 2.2 lp/mm with magnification) and Sanctorum et al. [75] (2.2 lp/mm and 2.5 lp/mm without magnification for the two image intensifiers and 3.4 lp/mm for magnification 1 and 4.6 lp/mm for magnification 2). Sanctorum et al. [75] used 4 megapixel cameras and recommend upgrading older systems to increase the resolution.

## Conclusion

We present an optimized, inexpensive, custom-made clamping technique that allows prolonged cyclic measurements of equine tendons. Repetitive cyclic testing of up to 50 min at 1.5 kN with Shetland pony SDFTs was successfully performed. Common problems such as tendon damage, disruption, or slipping did not occur. It was not necessary to suture or mechanically clamp the tendons. Loading of up to 10 kN was performed, which was not necessarily a physiological limit but the limit of the testing machine. Filling the clamp with liquid nitrogen via funnels enables repetitive cyclic measurements. Due to this cooling procedure and the mentioned technical adjustments of the clamp’s design, prolonged cyclic tensile testing was possible without any slippage or breaking of the tendon. We tested equine tendons, without ceasing, for up to 50 min, which represents a new precedent in tensile testing.

Tendon strain in motion was recorded with FluoKin by assessing changes of intermarker distances of implanted tantalum beads. Such a setup opens the way to monitor the segmental strain behaviour of tendons in long-term in vivo studies. The precision of bead tracking with videos derived from the FluoKin gait lab covers a range of ±0.1 mm (Tables 2 and 3). The precision of bead tracking in moving tendon tissue was 0.0553 mm — adding weight to our tensile results.

FluoKin is a useful experimental tool to assess the elasticity of insulted or healing tendons and to observe correlates of therapy success. Nevertheless, future in vivo investigations regarding tendon strain under different conditions, using FluoKin, are required and underway.

## Methods

### Specimens

From Shetland pony forelimbs, 17 SDFTs including the short pastern bone (os coronale) were dissected. These ponies had died of natural causes or were euthanized for medical reasons not related to this study. The forelimbs were provided after the veterinary post mortem examination. The maximum time of storage at − 20 °C before testing was 9 months. All specimens were examined with ultrasound (MyLabTwice, Esaote Biomedica Deutschland GmbH, Köln, Germany) to ensure only healthy tendons were tested. None of the specimens were discovered to be compromised. Additionally, the tendon diameter was measured (GE Healthcare, scil animal care company GmbH, Viernheim, Germany). Throughout all examination procedures the tendons were kept moist with isotonic saline solution. The dissected tendons were stored in double plastic bags at − 20 °C for a maximum of 16 days before testing and then thawed at 4 °C prior to the testing. To visualize the tendon’s length variation with FluoKin, 8 spherical tantalum beads (diameter of 0.8 mm, X-medics, Scandinavia, Frederiksberg, Denmark) were implanted into each tendon using an 18 G cannula and a custom-made mandrel controlled by ultrasound. Four beads were placed along the longitudinal axis in the tendon centre and supplemented with an additional spare bead at each level.

### Test protocol

**Fig. 6:**
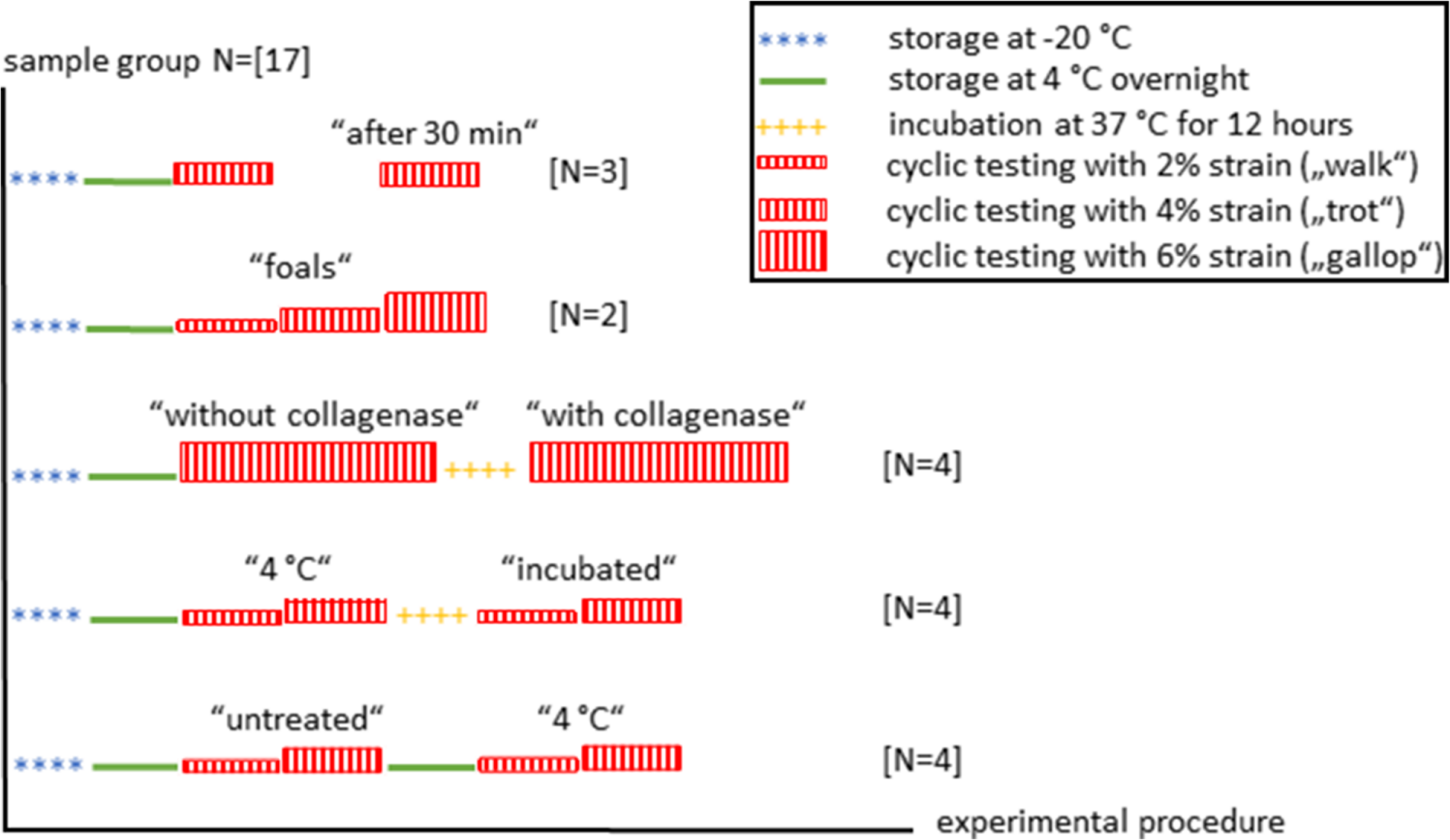
Schematic design for the testing protocol of each group

The tensile tests were performed with a Zwick Z 010® testing machine and its loading cell (< 10 kN) (ZwickRoell GmbH & Co. KG, Ulm, Germany). The speed of the machine was 1500 mm/min. Ten specimens were loaded with 2 and 4% strain to respectively simulate walk and trot ex vivo (Fig. 6). This was performed again with specimens which were stored at 4 °C overnight (referred to as the “4 °C” group) and incubated at 37 °C for 12 h (“incubated” group)*.* Two tendons from foals were loaded additionally with 6% strain (“foals” group). Four specimens received a 6% strain (“without collagenase” group) and were injected afterwards with 0.1 ml HBSS (Hank’s Balanced Salt Solution) and collagenase I (Life Technologies GmbH, Darmstadt, Germany, catalogue number 17100017) at a concentration of 4.8 mg/ml and incubated at 37 °C for 12 h (“with collagenase” group). Four additional specimens served as controls for the collagenase-treated group, also being incubated (control group “incubated”). For walk and trot respectively, in all conditions, strain was held for 0.2 s and 0.08 s (loaded holding phase) with an interval of 0.38 s and 0.24 s of rest between the loading cycles (unloaded phase). The specimens were continuously loaded with 70 loading/unloading cycles for up to 5 min and additionally video-radiographed with biplanar, high-speed, fluoroscopy after 60 loading cycles. Additionally, test protocols with 125 and 200 loading/unloading cycles or until tendon rupture were also carried out.

### Fixation device

The short pastern bone (os coronale) was mounted in a stainless-steel device that was screwed onto the loading machine via a 10 mm base plate (Fig. 4 a and b). The short pastern bone rested with its proximal articular surface on a bearing pin, where its curvature imitated the distal articular surface of the os coronale. Additionally, two adjusting gates with wheel screws held the bone in its position (Fig. 4 a and b). The SDFT was led across a rotatable pulley of 50 mm diameter to imitate the scutum proximale. The center-to-center distance of the bearing pin to the pulley was 67 mm. Due to their relative position to each other, the short pastern bone was mounted at an angle of approximately 50–55°.

The proximal end of the SDFT was fixed in a custom-designed cryogenic clamp made of aluminium (Fig. 4 c and d). The jaws were filled with liquid nitrogen via copper tubes and Teflon funnels. To achieve optimal cooling the clamping device was stored at − 80 °C overnight. From one solid piece, the two clamping jaws were cut apart in an optimized wave profile which allowed the converging of the jaws without creating shear forces. This occurs because at each distance of the jaws from one another, the space between the crest and trough of the wave is the same along the profile. At the upper and lower edges, the wave profile opens up by 10° and the edges are rounded with a radius of 2 mm to prevent disruption of the deep-frozen tendon. The 2.5 mm distance between wave crests was specifically adopted for pony SDFTs. Two holes were drilled vertically in each of the jaws, which were expanded with three horizontal recesses to enlarge the cooling surface (Fig. 4 e and d). One hole of each jaw was closed with a threaded plug with vent hole that could be opened if needed. The other hole was coated with rubber to hold the filling tube. The cryo-jaws were mounted to the testing machine with a u-shaped connector (Fig. 4 d).

### Biplanar high-speed fluoroscopy

FluoKin is as a 3D X-ray motion analysis system with biplanar arranged fluoroscopic units, which are retrofitted with high-speed cameras. A moving object is video-radiographed continuously for 6 s from two directions. This enables the reconstruction of its movements in three-dimensional space. To measure the length variation of soft tissue it is necessary to implant radiopaque markers, e.g. tantalum beads. The fluoroscopic examinations of the present study were performed at the FluoKin gait lab of Leipzig University, which is further described in Weiss et al. [77] and Geiger et al. [78]. Specimens were imaged at 46–50 kV and 80 mA, with 0.5 s shutter speed, without magnification, a source to image distance of 140 cm, and an interbeam angle of approximately 60° simulating an in vivo experimental set-up. Videos were recorded during the 60th testing cycle, at a resolution of 1024 × 1024 pixels, and at 200 fps. A sample video file showing the recording of one of the two high-speed cameras (during testing) can be found as Additional file 1. Undistortion, calibration, and marker tracking were performed with XMALab (version 1.3.8, www.bitbucket.org/xromm/xmalab, download 30-Sept 2016) according to Knoerlein et al. [79]. The tracked beads were manually adjusted after running an automatic marker tracking procedure as well as a polynomial fitting technique. The mean reprojection error was 0.2454 pixels. Data were filtered with a low-pass, 5th order Butterworth filter at 35 Hz.

### Precision measurements of the FluoKin system

Precision and accuracy were measured with A) pairs of steel and tantalum beads embedded, with a known distance, in an aluminium sheet (“sheet”) and B) tantalum beads implanted in a frozen distal pony forelimb. In a frozen object, intermarker distances should, ideally, not differ from frame to frame. Both test objects (A and B) were video-radiographed at 500 fps in a biplanar set-up of the gait lab. The imaging parameters deviated from those described above. The aluminium sheet was imaged at 50 and 55 kV and 100 mA, while the frozen distal forelimb was imaged with 55 and 63 kV and 100 mA. This ensured a satisfactory image quality. In the aluminium sheet, several kinds of beads had been embedded that are often used in our gait lab: 5 mm and 3 mm steel beads, 1.6 mm, 1 mm, and 0.8 mm tantalum beads. The distances of the embedded beads were determined via X-ray microtomography (Bruker Skyscan 1172–100-50). For the distal forelimb, five tantalum beads (diameter of 1.0 mm) were implanted into the metacarpal bone as widely distributed as possible while avoiding linearity. Four tantalum beads (diameter of 0.8 mm) were implanted into the SDFT. Both test objects were then video-radiographed statically (1976 frames of the sheet, 5473 frames of the forelimb) and dynamically by waving the sheet (2816 frames) and simulating a stepping motion with the forelimb attached to a wooden pole (5021 frames). Precision parameters were calculated as described by Brainerd et al. [18]. Additionally, the interior angles of all triangles between each combination of three bone or tendon beads, implanted in the frozen forelimb, were determined. There was no filtering of the data. Resolution of the FluoKin system was measured by imaging an X-ray test pattern. Statistical examination was carried out with t-tests using the RStudio software (RStudio Inc., Boston, Massachusetts, USA). Results were considered to be significant when *p* < 0.05.

## Supplementary Information

**Additional file 1.** Video of high-speed fluoroscopy during tensile testing. FluoKin video of one X-ray high-speed camera recorded during cyclic loading of the SDFT with implanted tantalum beads.

**Additional file 2.** Failure and cyclic tensile testing of Shetland pony SDFT. During the rupture tests of Shetland pony SDFTs, no slippage of the tendon tissue out of the cryo-clamps nor breakage of the tendon tissue above the clamps occurred. The shown repetitive cyclic testing protocol was carried out with 4% strain for repeated testing over at least 50 min.

## Data Availability

The datasets used and analysed during the current study are available from the corresponding author on reasonable request.

## Abbreviations

DDFT: Deep digital flexor tendon
FluoKin: Fluoroscopic kinematography, the 3 D X-ray motion analysis system at Leipzig University
SDFT: Superficial digital flexor tendon
DFT: Digital flexor tendons
