# Supplementary figures and images for: Cyclic tensile tests of Shetland pony superficial digital flexor tendons (SDFTs) with an optimized cryo-clamp combined with biplanar high-speed fluoroscopy
Source: BMC Vet Res. 2021 Jun 25;17:223. doi: 10.1186/s12917-021-02914-w (PMC8229380; doi:10.1186/s12917-021-02914-w)

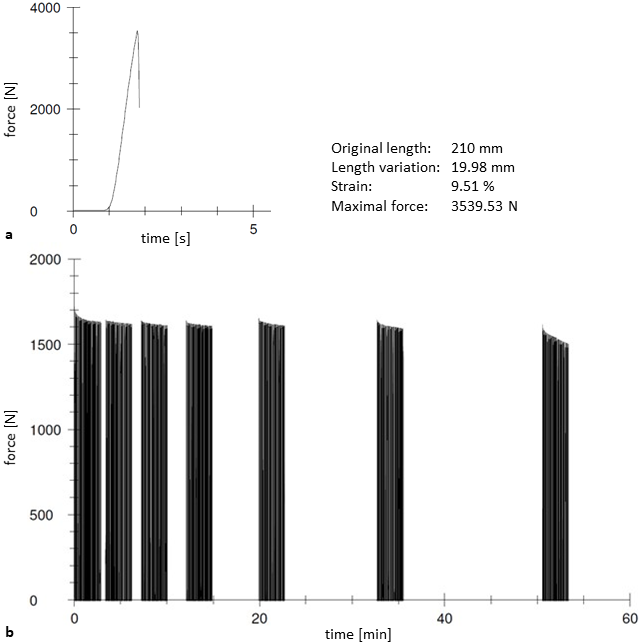

Supplement: Supplementary file 2 — Additional file 2. Failure and cyclic tensile testing of Shetland pony SDFT. During the rupture tests of Shetland pony SDFTs, no slippage of the tendon tissue out of the cryo-clamps nor breakage of the tendon tissue above the clamps occurred. The shown repetitive cyclic testing protocol was carried out with 4% strain for repeated testing over at least 50 min. [file 12917_2021_2914_MOESM2_ESM.png]
